# Genetic Mechanisms of Asthma and the Implications for Drug Repositioning

**DOI:** 10.3390/genes9050237

**Published:** 2018-05-03

**Authors:** Yue Huo, Hong-Yu Zhang

**Affiliations:** Hubei Key Laboratory of Agricultural Bioinformatics, College of Informatics, Huazhong Agricultural University, Wuhan 430070, China; huoyue@webmail.hzau.edu.cn

**Keywords:** asthma, disease-susceptible genes, drug repositioning

## Abstract

Asthma is a chronic disease that is caused by airway inflammation. The main features of asthma are airway hyperresponsiveness (AHR) and reversible airway obstruction. The disease is mainly managed using drug therapy. The current asthma drug treatments are divided into two categories, namely, anti-inflammatory drugs and bronchodilators. However, disease control in asthma patients is not very efficient because the pathogenesis of asthma is complicated, inducing factors that are varied, such as the differences between individual patients. In this paper, we delineate the genetic mechanisms of asthma, and present asthma-susceptible genes and genetic pharmacology in an attempt to find a diagnosis, early prevention, and treatment methods for asthma. Finally, we reposition some clinical drugs for asthma therapy, based on asthma genetics.

## 1. Introduction

Bronchial asthma is a chronic inflammatory disease of the respiratory tract, which involves a variety of cells and cellular components. Airway inflammation can cause airway hyperresponsiveness (AHR) and reversible airflow obstruction [1], which is manifested by repeated wheezing, chest tightness, cough or dyspnea, and asthma attacks that often occur at night or early in the morning. Asthma affects people of all ages, but it often occurs during early childhood and is the most common childhood respiratory disease.

In recent years, the morbidity and mortality rates of asthma have been constantly rising worldwide, and 1–18% of the populations of different countries are known to have asthma [2,3]. In China, the number of people suffering from asthma has increased to 30 million, including approximately 10 million children, and the prevalence of asthma among the urban residents is significantly higher than that in the rural areas [4]. With the increasing number of asthma patients, society and families bear the burden of medical investment and treatment costs. The recurrence of the disease and the aggravation of its symptoms affect the quality of life of patients. Currently, there is a preliminary understanding of the pathogenesis and predisposing factors of asthma, and a relatively mature diagnosis and treatment system has been established. However, genetic variability among asthmatic patients and the effects of varied environmental factors has led to differences in asthma pathogenesis and the efficacy of treatment [2,5]. This has motivated the analysis of the genetic mechanisms, susceptibility genes, and environmental factors that are underlying asthma, in order to provide a reference for the clinical treatment of asthma. With an understanding of the pathogenesis and the predisposing factors for asthma, and based on these asthma-susceptible genes, this paper focuses on finding potential asthma treatments, using a genetics-based drug repositioning method [6].

## 2. Pathogenesis of Asthma

Airway inflammation and airway remodeling are the physiological causes of asthma. Inflammation mainly occurs in the bronchi and conducting trachea, but may also spread proximally and distally across the trachea, even occurring near the alveoli with symptom aggravation [7]. Bronchial inflammation is closely associated with inflammatory cells (eosinophils, mast cells, T lymphocytes, neutrophils, macrophages, and airway epithelial cells) and structural cells (epithelial cells and smooth muscle cells). In addition, these cells produce mediators such as cytokines, chemokines, cysteine leukotrienes, and interferons, which can cause bronchial smooth muscle contraction and increase mucus secretion, further worsening the inflammatory milieu [8]. Reversible airway obstruction and airway hyperresponsiveness are major features of asthma [9]. Both airway inflammation and damage to the intraepithelial nerve can cause airway hyperresponsiveness, which is mainly manifested as bronchial swelling, thickening of the wall, and smooth muscle spasms, which further causes airway obstruction. In addition, the respiratory tract damage and its subsequent repair leads to airway remodeling, mainly subepithelial fibrosis; smooth muscle thickening; and basement membrane thickening. Airway remodeling makes irreversible changes to the respiratory tract structure and causes lung injury, making asthma treatment difficult [9,10].

## 3. Genetics of Asthma

Asthma is a complex disease with significant genetic predisposition and contributions [11,12]. So far, more than 100 asthma-related genes have been found [13]. By studying the polymorphisms of the susceptible genes, we can explain the heterogeneity of diseases and determine the asthma severity [14], which will aid in developing an appropriate treatment corresponding to a patient’s unique asthma pathogenesis.

### 3.1. Research Methods in Asthma Genetics

With the development of high-throughput sequencing and genotyping technology, and the improvement of computer data processing power, the ability to recognize genetic variation in asthma is increasing. There are three main genetic approaches to identifying asthma associated genes, namely, candidate gene association studies, positional cloning studies, and genome-wide association studies (GWAS). Most of the asthma genetic studies employ candidate gene association approaches, which have identified >50 genes in a number of studies [15]. The method is based on identifying the polymorphisms of the known functional genes that affect asthma. The method is limited in terms of understanding the pathobiological mechanisms of asthma and cannot detect new susceptible genes and pathways [12]. Positional cloning is based on the linkage analysis method for recognizing the chromosomal regions that are associated with the disease, and the disease-susceptible gene is identified by fine mapping. Using this method, six novel genes, which are closely related to asthma, have been identified, namely, *ADAM33*, *VDR*, *DPP10*, *PHF11*, *HLA-G*, and *GPR15* [16]. This method can also find asthma-susceptible genes in cases of an unknown gene function, but it is difficult to elucidate the complex disease mechanisms of asthma, as the method is restricted to a designated chromosomal region. With the improvement of high-throughput genotyping platform technology and the completion of the human genome project, GWAS with a higher accuracy are widely used, making it possible to exploit the common variants of complex diseases, including asthma. Nearly 100 asthma-related genes have been identified using GWAS, such as *IL33* on the 9p24 chromosomal region, *HLA-DR/DQ* on 6p21, *IL1RL1/IL18R1* on 2q12, and *IL13* on 5q31 [14].

### 3.2. Asthma-Susceptible Genes

Most of the asthma-susceptible genes that have been found so far are involved in the immune and inflammatory processes [13], which are also related to allergic diseases and airway hyperresponsive diseases, which reflects the association between asthma and other diseases at a genetic level [12]. It is generally believed that asthma-susceptible genes are mostly located in 5q31-33, 6p21, 12q13-q24, and other chromosomal regions [13], which have been determined by multiple experiments in different populations. We will focus on the genetic variants of the asthma-susceptible genes, and we will mainly describe the genetic features of asthma from the DNA level. We have collected the asthma-susceptible genes from several previous reviews [12,13,14,16,17,18,19], which have been reported in more than one study, and we also added some newly identified asthma-susceptible genes, in Table 1 [20]. The asthma-susceptible genes were classified into two categories, namely, those participating in an inflammation and immune response pathway, and those that are associated with airway structure and lung function. The following section will outline asthma-susceptible genes according to the two pathways (Table 1), which are classified and characterized according to the Database for Annotation, Visualization and Integrated Discovery (DAVID) bioinformatics resources and previous studies [21,22]. We have also listed the genetic variation in asthma-susceptible genes of different populations.

The inflammation and immune response pathway is particularly important in the pathogenesis of asthma. The human leukocyte antigen (HLA) plays a role in the regulation of inflammation in T helper cells [98]. The HLA class II molecules are involved in antigen presentation, and their polymorphism determines the presentation efficiency. This feature is closely related to the onset of asthma. The cytokines that are secreted by the inflammatory cells, including interleukins, chemokines, and tumor necrosis factor, are involved in triggering asthma and promote chronic airway inflammation [99]. These genes that encode cytokines are an important type of asthma-susceptible gene, and its polymorphism affects the severity of asthma. Targeted cytokine drugs are novel treatments for asthma. Toll-like receptors are pattern recognition receptor molecules that are located on the surface of airway epithelial cells, macrophages, and B cells. *TLR2*, *TLR4*, *TLR6*, *TLR9*, and *TLR10* polymorphisms are significantly associated with asthma risk [44].

With the asthma symptom aggravation, the development of inflammation increases the airway epithelial cell damage, which leads to airway hyperresponsiveness, airway remodeling, and lung injury. *ADAM33* is an asthma-susceptible gene, which has been identified by positional cloning studies, that is expressed in the airway stromal cells; it is involved in airway hyperresponsiveness and is associated with decreased lung function [7]. The *ORMDL3* gene at the 17q21 locus is thought to be closely associated with asthma, as it is expressed in a variety of inflammatory cells during the course of disease and it is associated with asthmatic airway remodeling [76].

Environmental risks factors are also one of the causes of asthma onset and aggravation. It is of great interest to elucidate the environmental risk factors in asthma attacks or the symptoms’ exacerbations, and the susceptible genes that are related to environmental factors in asthma. A combination of environmental variables and existing genomics data are the most commonly used research methods, including genome-wide interaction studies (GWISs), epigenome-wide association studies (EWAS) [77], GWAS data, and the encyclopedia of DNA elements (ENCODE) Project Set Data Integration Study [14]. Genome-wide expression profile studies [100] have been conducted to assess some asthma-susceptible genes that are related to smoke exposure, such as *ADAM33* [101], *TNF* [101], *GSTP1* [102], *GSTM1* [103], and *GSTT1* [103]. Indoor dust mites are common factors that induce and aggravate asthma symptoms. A study using genome-wide expression profiles has demonstrated that the interaction of *IL-9* genes with dust mites leads to the aggravation of asthma symptoms in children [100]. A variety of microbes that are prevalent in the environment and microbes’ components also induce asthma. For example, *TLR2* and *TLR4* genes are found to interact with endotoxin exposure factor, which is a generic term for the toxic substances that are produced by Gram-negative bacteria [104]. It can be seen from previous studies that environmental factors play a key role in asthma, but the systematic assessment of the interactions between genes and the environment is highly challenging. If we determine the mechanism of diseases that are induced by environmental factors and understand the impact of the different environmental factors on disease development [5], it could be possible to prevent asthma exacerbations and to guide the interventions of asthma.

Based on the asthma-susceptible genes, we can have a better understanding of this heterogeneous disease and apply a personalized treatment to asthma patients by distinguishing the genetic characteristics of different asthma phenotypes. Asthma has multiple phenotypes, and we have focused on the allergic and nonallergic asthma phenotypes in this paper. Allergic asthma is the major phenotype in asthmatic patients and nonallergic asthma occurs in about 10% to 33% of patients with asthma [105]. Nonallergic asthma has a later onset and higher degree of severity than allergic asthma [106]. In the clinical context, distinguishing the asthma phenotypes is difficult and the immunopathology is very complicated [7]. However, the distinct genetic profiles of asthma patients provide a new vision to distinguish the different asthma phenotypes. In previous studies, the −28C/G allele of the *CCL5* promoter region was uniquely associated with nonallergic asthma in the Japanese population [26], and a cluster research found that the polymorphism in *SRP9* (rs4653433) was related to nonallergic asthma [17]. Furthermore, the polymorphism in *Il1RL1*, *SMAD3*, *RORA*, *ORMAL3*, *DPP10*, *TSLP*, *IL13*, *HLA-DQ*, *IL12RB*, and *HRH1* was significantly associated with the allergic asthma phenotype [17]. In particular, the genetic variation along the histamine pathway is unique in allergic asthma versus nonallergic asthma [107].

### 3.3. Pharmacogenetics of Asthma

Finding asthma-susceptible genes is a key step towards uncovering the pathogenesis of asthma, but it is more important to know how the genetic variations and drug therapy work together. Pharmacogenetics mainly studies the genetic variation of individuals with regard to drug treatment responses. By mining the genes related to drug reactions, it is possible to develop individualized treatment regimens for patients and to achieve precise medications, minimize drug side effects, and improve treatment outcomes. The genetic pharmacology asthma studies have found that the genetic variation affects drug responses through different mechanisms, including some pharmacokinetic mechanisms that are induced by receptor agonists, as well as others that determine the drug metabolism [2]. Here, we have mainly reviewed the pharmacogenetics of glucocorticoids, β2-adrenergic agonists, and leukotrienes. As a result of the heterogeneity of this disease, the different genetic variant that has caused an inconsistent drug treatment response in different populations and the partial studies, has not been reproduced in more populations, so we should consider the association between the genetic variants and their corresponding study populations to the association between the genetic variants and the corresponding study population.

Glucocorticoids (GCs) are common anti-inflammatory drugs that are used for asthma therapy. The inhaled corticosteroids can effectively improve the symptoms of asthma and reduce the disease exacerbation, however long-term high doses can cause endocrine disorders, hypoimmunity, and other side effects. The genetic pharmacology of glucocorticoids in asthma is generally associated with pulmonary function, airway responsiveness, and disease exacerbation [108]. Studies have shown that the protein that is encoded by the *STIP1* gene is an important component of the complex that activates the glucocorticoid receptor. The *STIP1* gene polymorphism affects the therapeutic effect of steroids [109]. *TBX21* encodes T-bet, which is necessary for naive T-lymphocyte production. The patients with a common nonsynonymous single nucleotide polymorphism (SNP) in *TBX21*, had significantly improved airway hyperresponsiveness after taking glucocorticoids, as demonstrated in children [108]. *CRHR1* encodes a G-protein coupled receptor that binds to the neuropeptides that are associated with corticotrophin release and participates in the regulation of the hypothalamic-pituitary-adrenal pathway, which improved the lung function response to glucocorticoids in three populations [5]. The *FCER2* gene encodes a low affinity IgE receptor, and a new variant (rs28364072) can increase the severity of asthma after using glucocorticoids, which has been identified by Childhood Asthma Management Program, as demonstrated in children [110].

The short-acting and long-acting β2-adrenoceptor agonists are the most commonly used bronchial dilatation prescription medications, but long-term use can cause heart rate disorders and bronchial injury. *ADRB2* is a β2-adrenergic receptor gene, and the polymorphism at this locus affects the efficacy of asthma therapeutics. The mutation of the Arg16 homozygote of the *ADRB2* gene weakens the pharmacological response to the short-acting β agonists in several experiments [5]. Steroid-resistant asthma takes the majority of health care budget that is dedicated to asthma, and the glucocorticoid receptor gene variants may cause steroid resistance. A case-control study indicated that the D641V variant of the glutathione reductase *(GR)* gene is related to steroid-resistant asthma, in the Chinese Han population [111].

Leukotriene drugs include cysteinyl leukotriene receptor antagonists and 5-lipoxygenase inhibitors, which have good safety ratings, are amenable to long-term use, have minimal side effects, and can significantly improve the lung function of asthma patients. However, the polymorphism of some genes leads to differences in the therapeutic effect of leukotrienes. *ALOX5* is a major target for leukotrienes, and studies have shown that the mutations in the *ALOX5* promoter region affect the leukotriene therapeutic effect. The genetic variation of the target provides a reference for the diagnosis and administration for asthma [112]. A clinical trial in white and African American populations has demonstrated that the variation of the A444C SNP in the leukotriene C4 synthase gene in the leukotriene metabolic pathway and the SNP variation in intron 2, which encoded the *LTA4* hydrolase gene, had cause differential responses to leukotriene receptor antagonists [65].

## 4. Drug Repositioning Based on Genetics

In clinical practice, drug therapy is still the most effective means of preventing exacerbations and for the treatment for asthma. The asthma drugs are mainly divided into two types, namely, bronchodilators (e.g., β2-adrenoceptor agonists, anticholinergics, and theophylline drugs) and anti-inflammatory drugs (e.g., glucocorticoid, anti-allergic drugs, and leukotrienes). Although there are various asthma drugs on the market, there are a large number of asthma patients and the current drugs are not working in a significant proportion of people with asthma, suggesting that the development of new drugs is necessary. The current drug research and development funding is increasing, with the advance of high throughput sequencing technologies. However, new drug research and development efficiency is a great challenge. In this paper, we use a genetics-based drug repositioning method to find new drugs that have potentially therapeutic effects for asthma patients [6,113].

Disease-susceptible genes are an important drug target source [2]. However, not all asthma-susceptible genes are suitable drug targets. Genes that are strongly correlated with the disease phenotypes and that have been reported in multiple studies, are called top genes, which implies that these genes have a higher possibility as drug targets [106]. According to the relevant reviews [13,19], we have collected 34 genes, which have been reported in more than five related studies as top genes. We have considered that these genes are more druggable than others. Furthermore, the targets for the approved asthma drugs must have stronger gene-disease correlations and also can be used for drug repositioning.

Normally, an ideal target contains a series of functional alleles related to the disease phenotypes, which can lead to loss of function (LOF) or gain of function (GOF) of genes, which is caused by certain genetic variants [114,115]. The LOF or GOF of genes may lead to asthma attacks, and the agonists or antagonists that target these genes are potential therapeutic agents [6,113], respectively. Thus, an understanding of the drug action modes and the pathogenesis of genetic diseases helps us to find novel anti-asthma drugs, based on genetics. As shown in Table 2, many typically approved asthma drugs meet these target-drug correspondence criteria, demonstrating the feasibility of this method for the discovery of anti-asthma medications. By combining the information for the asthma top genes, asthma druggable genes, and drug modes of action, we have found several potential anti-asthma medications from drugs for other diseases, which have not been approved for the treatment of asthma (Table 3). The asthma genetics and asthma-related genes are the basis of drug repositioning, and we have also combined with the asthma phenotype in order to increase the efficiency of drug repositioning and to provide guidance for asthma treatment.

The asthma-related genes were the basis of drug repositioning, and we also combined them with the role of these targets in the asthma pathogenesis and asthma phenotype, in order to increase the efficiency of the drug repositioning and to provide guidance for asthma treatment. Cytokines played an important role in the asthmatic inflammatory response. Consequently, the drugs that targeted cytokines were hotspots for the development of new anti-asthma drugs, which provided a new insight into personalized medicine for different asthma phenotypes [14]. For example, there were several approved biological drugs that targeted the cytokines for asthma treatment, Mepolizumab, Omalizumab, Reslizumab [132]. In mild and moderate asthma, the T helper type 2 (Th2) cells dominated over the T cell lineage in the airway, which were the producers of the typeII cytokines IL4 and IL13, which had a high message and protein level in asthma patients [133]. The IL4 Interleukin participated in the Th2 cell differentiation and suppressed the T helper type 1 (Th1) cell development, and also contributed to the eosinophil recruitment and IgE synthesis [133]. IL13 promoted the IgE production, release eosinophil chemoattractants and increasing mucus secretion, which caused the bronchial smooth muscle contraction and epithelial fibrosis. This led to the occurrence of airway hyperresponsiveness, which played a crucial role in the asthma pathological features [133,134]. Both IL4 and IL13 were important in asthma pathology, typically in those patients with Th2 profile inflammation. The inhibitors that targeted IL4RA could potentially block the IL4/IL13 signal pathway. The combined approach to weaken the effects of IL4/IL13 was more effective in asthma therapy anti-IL4/IL13 drugs and had been mainly applied in cases of mild atopic asthma and as an additional treatment, based on the inhaled corticosteroids plus a long-acting β2 agonist, in patients with uncontrolled persistent asthma [122,135]. Recently, some studies have showed that dupilumab, a new anti-IL4/IL13 approach, could decrease asthma exacerbation and improve lung function [122]. However, this therapy also needed to be used in combination with the inhibition of eosinophil inflammation [99]. As a result of the lack of the replicate studies in several populations, the safety and effectiveness of dupilumab could not be fully evaluated [122]. Consequently, clinical experiments and a security assessment of dupilumab are necessary.

With asthma exacerbation, Th1 T cells were recruited and they secreted TNF-α and interferon [8]. The levels of the TNF-α protein and messenger RNA (mRNA) in patients with severe asthma were elevated, and the TNF-α promoted airway inflammation and airway hyperresponsiveness (AHR), which play a central role in airway remodeling. TNF-α was a chemical inducer of neutrophils and eosinophils. It was involved in the activation of T cells and promoted the transfer of inflammatory cells to the lungs. In severe asthma, TNF-α also recruited neutrophils, induced resistance to glucocorticoids, and stimulated fibroblast growth [136]. *TNF-α* was an important pleiotropic cytokine in patients with asthma, and played a key role in airways hyperresponsiveness and other asthma features. Anti-TNF-α drugs could improve lung function, airway hyperresponsiveness, and reduce exacerbation frequency in patients with severe asthma [137]. The related studies and investigators have shown that anti-TNF-α drugs are beneficial for the subset of patients who have a high TNF-α level in airways with severe asthma [137]. There have been some clinical trials for *TNF-α* biological inhibitors, for example, infliximab, etanercept, and adalimumab, which have different mechanism of action. Infliximab is a monoclonal antibody which prevents TNF-α from interacting with its receptors. Etanercept binds specifically to tumor necrosis factor (TNF) and modulates related biological processes regulated by TNF. Adalimumab also can bind specifically to TNF-α and reduce TNF-induced inflammation and halting tissue destruction [118]. A trial had demonstrated that etanercept could improve the symptoms of asthma patients with severe corticosteroid dependent asthma [123], and a study also found that the treatment of etanercept resulted in an improvement in the asthma-related quality-of-life score in mild-to-moderate asthma patients [124]. However, the etanercept had no obvious therapy effects in another study [138]. The treatment of infliximab could decrease the level of TNF-α and improve the lung function of moderate asthma patients and were well tolerated [125,126]. An experimental study found that adalimumab could reduce the lung damage in a murine model with acute asthma. According to the above clinical trials, the overall efficacy of these drugs in the treatment of asthma was modest and the safety of anti-TNF-α drugs was uncertain [126]. The anti-TNF-α drugs were still controversial for the treatment of asthma, the application of treatment for asthma should have been done more carefully, and this therapy should have been used for particular populations. Consequently, further large samples, multiple population experiments, and longer term follow-up experiments would be needed in order to verify the efficacy and the side effects of the *TNF-α* inhibitors. In addition, the application of the anti-TNF-α drugs that were aimed at the differential asthma phenotype and the right treatment prescription were significate in asthma therapy.

Histamine played an important role in the process of asthma inflammation, which led to the increased vascular permeability, mucus secretion, and airway smooth muscle cell contraction. The levels of *HRH1* mRNA were significantly elevated in asthmatic patients, and the dose effects were associated with the asthma severity [53]. Furthermore, the generic variation within the histamine pathway was associated with allergic asthma [107]. In the seasonal allergic rhinitis that was associated with asthma, Desloratadine could relieve the symptoms of seasonal allergic rhinitis, and reduce the daily β2-agonists dose [128]. Cysteine leukotriene was also an important mediator of asthma, which depended on the release of arachidonic acid and the activation of 5-lipoxygenase (*ALOX-5*). In addition, the Leukotriene modifying drugs were used as the additional therapy in severe asthma and as the primary therapy for childhood asthma [14,65]. The cysteinyl leukotriene receptor 1 (*CYSLTR1*) antagonists and the inhibitor of *ALOX-5* were the main drugs that were used to block the effect of cysteinyl leukotriene in asthma [14]. Therefore, the *CYSLTR1* and *ALOX-5* were the meaningful target for drug repositioning.

In short, based on the asthma-susceptible genes, some drugs could potentially be repositioned for asthma therapy (Table 3). Many of the predicted drugs had been validated by experimental evaluation in a part of populations, which supported the potential effectiveness of the genetics-based drug repositioning method. However, these drugs had not been reproduced in larger cohorts, and we had not found any report of these repositioned drugs, such as lonapalene, flobufen, masoprocol, LY-2300559, and ixekizumab, for asthma treatment.

## 5. Conclusions

Asthma is an incurable chronic bronchial disease. Drug treatment is designed to control the progression of the disease and reduce the number of episodes. However, the complicated pathogenesis of asthma, heterogeneity of patients, side effects of drugs, poor drug compliance, and other causes has led to poor asthma control. Thanks to the development of genomics and genetics, we could discover the underlying susceptible genes in order to provide a theoretical basis for the discovery of new drug targets and precision medications. Epigenetics and related studies focus on the key environmental factors that are related to asthma genetics, guiding the early prevention of asthma and disease control. In addition, through observing the individual differences in response to asthma drugs and by analyzing the underlying genetic pharmacological mechanisms, we could develop a personalized approach to the diagnosis and treatment of patients. Although the overall rate of drug discovery is currently declining, this process may be facilitated through genetics-based drug repositioning. However, there is still a long way to go regarding asthma treatment. Starting from the early stage of the disease diagnosis and drug treatment, we need to pay more attention to each patient’s individual differences at each stage of treatment so as to best control the patient’s condition.

## Figures and Tables

**Table 1:** Asthma-susceptible genes and classification.

Categories	Gene	Chromosome ^1^	Molecular Function ^2^	Variants	Population	References
**Inflammation and Immune Response Pathway**						
**Cytokines**						
	*CC16*	11q12.3	secretoglobin family 1A member 1	38A/G	Japanese	[23]
	*CCL11*	17q12	CCR chemokine receptor binding	−384A/G	African American	[24]
	*CCL5*	17q12	phosphatidylinositol phospholipase C activity; chemokine receptor binding	−403G/A −28C/G	Multiple countries Japanese	[25,26]
	*CSF2*	5q31.1	colony-stimulating factor receptor binding;	rs25882	Swiss	[27]
	*IFNG*	12q15	interferon-gamma receptor binding;	874A/T	Chinese Han	[28]
	*IL10*	1q32.1	interleukin-10 receptor binding;	−1082A/G	East Asians	[29]
	*IL12B*	5q33.3	cytokine receptor activity	rs3212227	n.a. ^3^	[30]
	*IL13*	5q31.1	interleukin-13 receptor binding	rs20541 rs848 rs1295686	Multiple countries Italy Multiple countries	[20,31,32]
	*IL1B*	2q14.1	interleukin-1 receptor binding	rs16944 rs1143634	n.a. ^3^	[30]
	*IL33*	9p24.1	protein binding	rs928413 rs3939286 rs1342326	Multiple countries Multiple countries Dutch	[17,32,33]
	*IL4*	5q31.1	interleukin-4 receptor binding; growth factor activity	−589C/T 33C/T	Europeans; Iranian	[34,35]
	*IL5*	5q31.1	interleukin-5 receptor binding; growth factor activity	−703C/T	Russia	[36]
	*IL6*	7p15.3	interleukin-6 receptor binding; growth factor activity	−174G/C (rs1800795)	Multiple countries	[37]
	*LTA*	6p21.33	tumor necrosis factor receptor binding	NcoI	Multiple countries	[38]
	*MIF*	22q11.23	cytokine receptor binding	−173G/C	Korea; Egyptian	[39,40]
	*STAT6*	12q13.3	nucleic acid binding; transcription factor; DNA binding transcription factor activity and sequence-specific DNA binding	rs167769 rs71802646	Multiple countries	[20,41]
	*TNF*	6p21.33	tumor necrosis factor receptor binding	−308G/A −238G/A	n.a. ^3^	[30]
	*TSLP*	5q22.1	cytokine activity	rs1837253 rs3806933 rs2289276	Multiple countries Japanese	[17,42]
**Toll-like receptors**						
	*TLR10*	4p14	transmembrane signaling receptor activity	2322A/G 1031G/A	African American; European American	[43]
	*TLR2*	4q31.3	Toll-like receptor binding	Arg753Gln	Multiple countries	[44]
	*TLR4*	9q33.1	transmembrane signaling receptor activity	Asp299Gly Thr399Ile	Multiple countries	[44]
	*TLR6*	4p14	toll-like receptor 2 binding; transmembrane signaling receptor activity	Ser249Pro	African Americans; European Americans; Hispanic Americans	[45]
	*TLR9*	3p21.2	interleukin-1 receptor binding; transmembrane signaling receptor activity	−1237T/C	Multiple countries	[44]
**Major histocompatibility complexes**						
	*HLA-DP*	6p21.32	MHC class II receptor activity	rs987870	Asian	[46]
	*HLA-DQA1*	6p21.32	MHC class II receptor activity	rs9272346	Multiple countries	[47]
	*HLA-DQB1*	6p21.32	MHC class II receptor activity	rs9273349	Multiple countries	[32,47]
	*HLA-DRB1*	6p21.32	MHC class II receptor activity	rs9272346 rs9271300	Multiple countries Australian	[20,48]
**Receptors**						
	*CD14*	5q31.3	opsonin receptor activity	−260C/T	Korean;French	[49,50]
	*FCER1B*	1q23.2	IgE receptor activity	−109C/T	Indian; Chinese; Japanese	[51,52]
	*HRH1*	3p25.3	G-protein coupled receptor activity; histamine receptor activity	−17T/C (rs901865)	Multiple countries	[53]
	*IL12RB*	19p13.11	cytokine activity	rs2284033	Multiple countries	[32]
	*IL18R1*	2q12.1	interleukin-18 receptor activity	rs3771166	Multiple countries	[32]
	*IL1RL1*	2q12.1	cytokine receptor activity; receptor signaling protein activity	rs17026974 rs13431828 rs1420101	Multiple countries	[17,20]
	*IL4RA*	16p12.1	cytokine receptor activity; signal transducer activity, downstream of receptor	rs1805011	Caucasians	[54]
	*IL5RA*	3p26.2	interleukin-5 receptor activity	5993A/G	Korean	[55]
	*IL6R*	1q21.3	interleukin-6 receptor activity	−174C/G	Finnish	[56]
	*PTGDR*	14q22.1	prostaglandin D receptor activity	−731A/G 6651C/T	Caucasian	[57,58]
	*TBXA2R*	19p13.3	thromboxane A2 receptor activity	795C/T	Multiple countries	[59,60]
				−924C/T		
				rs8113232	n.a. ^3^	
**Cysteine leukotriene metabolic pathway**						
	*ALOX5*	17p13.2	arachidonate 5-lipoxygenase activity	rs59439148	African American; White	[61]
	*CYSLTR1*	Xq21.1	cysteinyl leukotriene receptor activity	rs2637204	Japanese	[62]
	*CYSLTR2*	13q14.2	cysteinyl leukotriene receptor activity	−1220A/C −819T/G	Japanese Koreans	[63,64]
	*LTC4S*	5q35.3	leukotriene-C4 synthase activity	−444A/C	African American; White	[61,65]
**Airway hyperresponsiveness, airway remodeling, lung function**						
	*ACE*	17q23.3	actin binding; drug binding	I/D polymorphism	Multiple countries	[66]
	*ADAM33*	20p13	metalloendopeptidase activity; zinc ion binding	rs528557	Multiple countries	[67,68]
	*ADRB2*	5q32	beta2-adrenergic receptor activity	Arg16Gly Gln27Glu	Multiple countries	[69,70]
	*AREG*	4q13.3	epidermal growth factor receptor binding; cytokine activity	rs204993	Japanese	[71]
	*CHI3L1*	1q32.1	hydrolyzing O-glycosyl compounds; carbohydrate binding; hydrolase activity	rs4950928 rs12141494	European	[72]
	*DPP10*	2q14.1	dipeptidyl-peptidase activity	rs10208402 rs1435879	Chinese	[73,74]
	*NOS1*	12q24.22	nitric-oxide synthase activity	3391C/T 5266C/T	Chinese	[75]
	*ORMDL3*	17q21.1	protein binding	rs8076131 rs12603332 rs7216389	Multiple countries	[17,76,77]
	*PLAU*	10q22.2	serine-type endopeptidase activity; protein binding; kinase activity	rs2227564C rs2227566C	French-Canadian familial	[78]
	*SERPINB4*	18q21.33	serine-type endopeptidase activity; protease binding	n.a. ^3^		[18]
	*SERPINE1*	7q22.1	serine-type endopeptidase activity; protease binding	−675 4G/5G	Dutch	[79]
	*SERPINH1*	11q13.5	serine-type endopeptidase activity; protease binding	n.a. ^3^		[18]
	*TGFB1*	19q13.2	cytokine activity; transforming growth factor beta receptor binding	−509C/T	White	[19,80]
	*CMA1*	14q12	endopeptidase activity	−1903G/A	Egyptian children	[81]
**Others**						
	*BACH2*	6q15	transcription factor activity; sequence-specific DNA binding	rs2325291	Multiple countries	[20]
	*CLEC16A*	16p13.13	protein binding	rs17806299	Multiple countries	[20]
	*CRHR1*	17q21.31	G-protein coupled receptor activity	rs242941	Indian children	[82]
	*EMSY*	11q13.5	protein binding; protein homodimerization activity	rs7927894	Multiple countries	[20]
	*ERBB2*	17q12	protein binding; signal transducer activity	rs2952156	Multiple countries	[20]
	*FLG*	1q21.3	structural molecule activity; protein binding	2282del4	White; Danish	[83,84]
	*GATA3*	10p14	DNA binding transcription factor activity; transcription factor binding	rs2589561	Multiple countries	[20]
	*GPRA*	7p14.3	neuropeptide S receptor 1	rs324384 rs324396	Western European	[85]
	*GPX5*	6p22.1	glutathione peroxidase activity	rs1233578	Multiple countries	[20]
	*GSTM1*	1p13.3	glutathione transferase activity; transferase activity; enzyme binding	+/null	Multiple countries	[86]
	*GSTP1*	11q13.2	glutathione transferase activity	IIe105Val Ala114Val	Multiple countries	[87]
	*GSTT1*	22q11.23	glutathione transferase activity	A/null	Caucasians	[88]
	*ITGB3*	17q21.32	receptor activity	rs3809865	Multiple countries	[89,90]
	*MICB*	6p21.33	natural killer cell lectin-like receptor binding	rs2855812	Multiple countries	[20]
	*NAT2*	8p22	arylamine *N*-acetyltransferase activity	Acetylation genotypes	Caucasians	[91]
	*NDFIP1*	5q31.3	protein binding; signal transducer activity	rs7705042	Multiple countries	[20]
	*NOD1*	7p14.3	protein binding; cysteine-type endopeptidase activator activity involved in apoptotic process	ND (1) + 32656	Australia; America	[92]
	*RANBP6*	9p24.1	transporter activity; protein binding	rs992969	Multiple countries	[20]
	*RORA*	15q22.2	DNA binding transcription factor activity and transcription factor binding	rs11071558 rs11071559	Multiple countries	[20,32]
	*SLC25A46*	5q22.1	protein binding	rs10455025	Multiple countries	[20]
	*SMAD3*	15q23	sequence-specific DNA binding transcription factor activity	rs744910 rs2033784	Multiple countries	[32]
	*SPINK5*	5q32	serine-type endopeptidase inhibitor activity	Glu420Lys	German	[93]
	*SRP9*	1q42.12	RNA binding; protein binding	rs4653433	Multiple countries	[94]
	*TPD52*	8q21.13	protein binding	rs12543811	Multiple countries	[20]
	*VDR*	12q13.11	steroid hormone receptor activity; vitamin D response element binding	TaqI, BsmI, and FokI polymorphisms	Multiple countries	[95,96]
	*ZNF652*	17q21.32-q21.33	nucleic acid binding; DNA binding; protein binding	rs17637472	Multiple countries	[20]

^1^ Derived from NCBI Gene (https://www.ncbi.nlm.nih.gov/gene/); ^2^ derived from DAVID [21,22], GeneCards [97]; ^3^ Not available (n.a.). CCR: C-C motif chemokine receptor MHC: major histocompatibility complex.

**Table 2:** Correspondence between target pathogenesis and drug mode of action for approved asthma-therapeutic drug.

Target	Pathogenesis ^1^	Drug (Mode of Action) ^2^	Side Effects ^3^
*GR*	GOF	Budesonide (antagonist)	Motor activity, piloerection, generalized edema;
*CYSLTR1*	GOF	Montelukast (antagonist)	Headache, abdominal or stomach pain, cough, dental pain, dizziness, fever, heartburn, skin rash, stuffy nose, weakness or unusual tiredness;
*CYSLTR1*	GOF	Pranlukast (antagonist)	Headache, abdominal or stomach pain, cough, dental pain, dizziness, fever, heartburn, skin rash, stuffy nose, weakness or unusual tiredness;
*HRH1*	GOF	Chloropyramine (antagonist)	Agitation and dizziness;
*HRH1*	GOF	Emedastine (antagonist)	Somnolence and malaise;
*IL-5*	GOF	Mepolizumab (antagonist)	Blurred, confusion, cough, difficulty with breathing, dizziness, noisy breathing, sweating, tightness in the chest, swelling, hives, blisters and tiredness;
*IL-5*	GOF	Reslizumab (antagonist)	Allergic reactions, anaphylaxis, cancer, muscle pain;
*ADRB2*	LOF	Isoetarine (agonist)	Tachycardia, palpitations, nausea, headache, and epinephrine-like;
*ADRB2*	LOF	Salbutamol (agonist)	Tremor, hypersensitivity reaction and tachycardia;

^1^ Derived from Online Mendelian Inheritance in Man (OMIM) [116]. GOF: gain of function; LOF: lose of function; ^2^ Derived from Therapeutic Targets Database (TTD) [117], DrugBank [118] and ClinicalTrials [119]; ^3^ Derived from DrugBank [118], Drugs.com [120] and The Medical Dictionary [121].

**Table 3:** Genetics-based drug repositioning for asthma therapy.

Target	Pathogenesis ^1^	Drug (Mode of Action) ^2^	Current Drug Indication ^2^	Source ^3^	Reference ^4^
*IL4RA*	GOF	Dupilumab (antagonist)	Atopic dermatitis	Top gene	[1,122]
*TNF-α*	GOF	Etanercept (antibody)	Psoriasis;	Top gene	[123,124]
*TNF-α*	GOF	Infliximab (inhibitor)	Psoriasis; Crohn’s disease;	Top gene	[125,126]
			Ankylosing spondylitis;		
			Psoriatic arthritis; Rheumatoid arthritis; Ulcerative colitis		
*TNF-α*	GOF	Adalimumab (antibody)	Ankylosing spondylitis;	Top gene	[127]
			Rheumatoid arthritis		
*HRH1* *HRH1*	GOF GOF	Desloratadine (antagonist) Mepyramine maleate (antagonist)	Allergic rhinitis Allergy	Druggable gene Druggable gene	[128,129,130] [131]
*Alox-5* *Alox-5* *Alox-5*	GOF GOF GOF	Lonapalene (inhibitor) Flobufen (inhibitor) Masoprocol (inhibitor)	Psoriasis Rheumatold arthritis Prostate cancer	Druggable gene Druggable gene Druggable gene	n.a. n.a. n.a.
*CYSLTR1*	GOF	LY-2300559 (antagonist)	Migraine	Druggable gene	n.a.

^1^ Derived from OMIM [116]. ^2^ Derived from TTD [117], DrugBank [118], and ClinicalTrials [119]. ^3^ Top genes were derived from some previous literatures [11,67], and druggable genes were derived from DrugBank. ^4^ References supporting the potential therapy on asthma. n.a not available. TNF: tumor necrosis factor, IL: interleukin.
